# Supplementary material for: Exoproducts of the Most Common Achromobacter Species in Cystic Fibrosis Evoke Similar Inflammatory Responses In Vitro
Source: Microbiol Spectr. 2023 Jun 7;11(4):e00195-23. doi: 10.1128/spectrum.00195-23 (PMC10434066; doi:10.1128/spectrum.00195-23)
Supplement: Supplemental file 2 — Legends of Fig. S1 to S4. Download spectrum.00195-23-s0001.docx, DOCX file, 0.03 MB [file spectrum.00195-23-s0001.docx]

**Figure legends Supplement**

*Supplementary figure 1: Pilot studies for the titration of bacterial supernatant concentration. Supernatants from the* A. xylosoxidans *type strain CCUG-56438T was used for titration experiments. Upon stimulating BEAS-2B lung epithelial cells (A), a concentration of 5% gave rise to nearly as strong IL-6 secretion as 50%, and even a slightly higher release of IL-8. LB medium alone did not give rise to any background signal in this experiment. When stimulating neutrophils and monocytes, 5% supernatant was the lowest concentration used. Upregulation of CD11b was analysed to assess leukocyte activation using flow cytometry. After an incubation time of 2 hours, 5% supernatant caused similar neutrophil (B) and monocyte (C) activation as the positive control fMLF.*

*Supplementary figure 2: Light microscopy images of CFBEo- cells to estimate cell health. Upon visual inspection, there was no observable difference between wells exposed to (A) Achromobacter supernatants (*A. xylosoxidans*), (B) Pseudomonas supernatant (PAO1) or (C) negative controls. These images have been selected as representative of cell appearance as it did not differ noticeably between any of the isolates.*

Supplementary figure 3: Cytokine responses from CFBEo- cell cultures stimulated with 5% (v/v) bacterial supernatant. The Figure shows concentrations of IL-6 (A) and IL-8 (B) in the cell culture medium after 24h of stimulation. Each symbol represents a bacterial isolate and shows the mean value of three independent replicates. Bars illustrate the mean. Pairwise comparisons were made using Mann-Whitney U test (* = p < 0.05).

*Supplementary figure 4: Leucocyte activation by stimulation with 5% (v/v) bacterial supernatant in whole blood. CD11b expression (A, B) and HBP release (C) are calculated as fold increase over negative control (HEPES), with no increase compared to negative control labelled as a line (1-fold). fMLF is used as positive control. All supernatants caused significant increase of CD11b expression in neutrophils (A) and monocytes (B) compared to LB. The supernatants of all species studied gave rise to a significant HBP release compared to LB (C). Each dot represents one bacterial isolate or control as the average value of 3 repeats. Bars represent the mean with SD. Pairwise comparisons were made using Mann-Whitney U test (* = p* < 0.05)*.*
