# Supplementary figures and images for: Exoproducts of the Most Common Achromobacter Species in Cystic Fibrosis Evoke Similar Inflammatory Responses In Vitro
Source: Microbiol Spectr. 2023 Jun 7;11(4):e00195-23. doi: 10.1128/spectrum.00195-23 (PMC10434066; doi:10.1128/spectrum.00195-23)

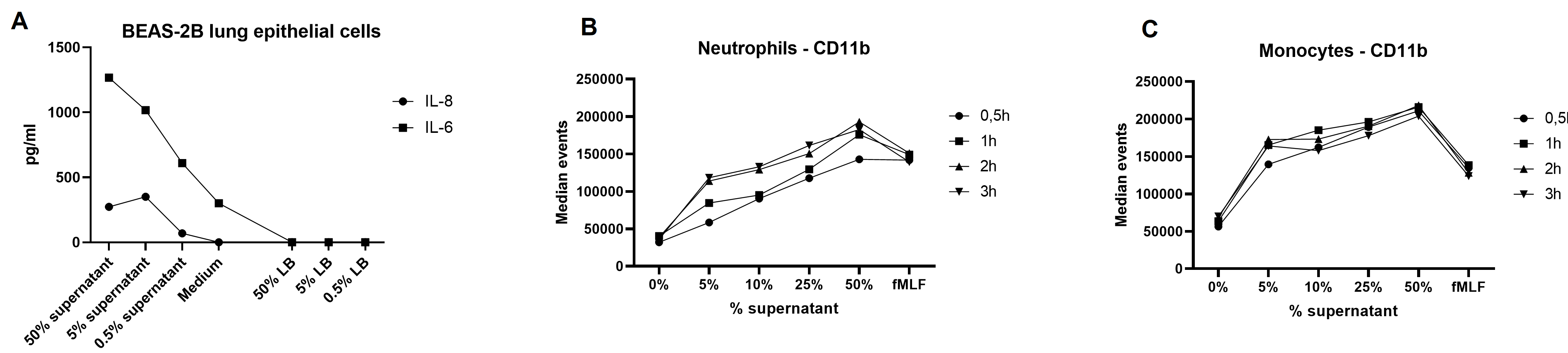

Supplement: Supplemental file 3 — Fig. S1. Download spectrum.00195-23-s0002.tif, TIF file, 0.2 MB [file spectrum.00195-23-s0002.tif]

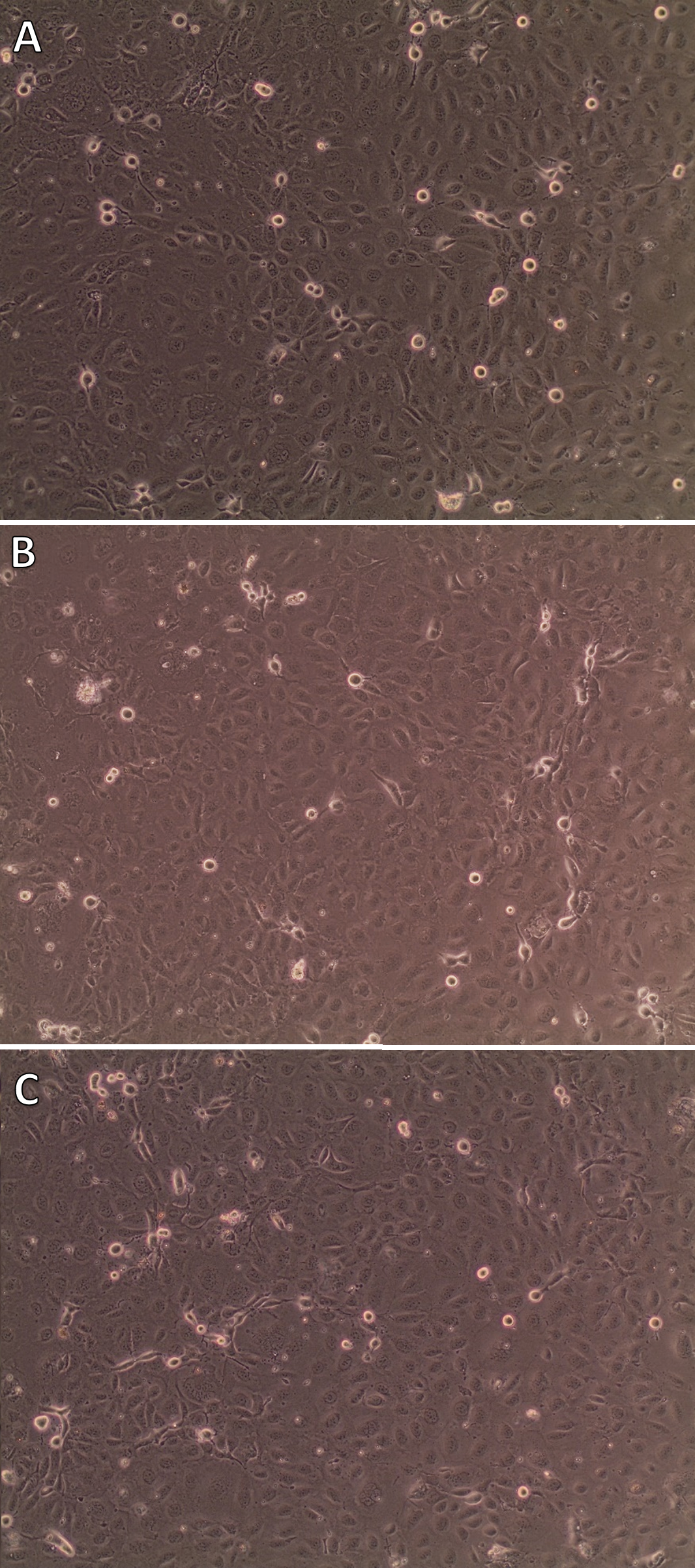

Supplement: Supplemental file 4 — Fig. S2. Download spectrum.00195-23-s0003.tif, TIF file, 3.9 MB [file spectrum.00195-23-s0003.tif]

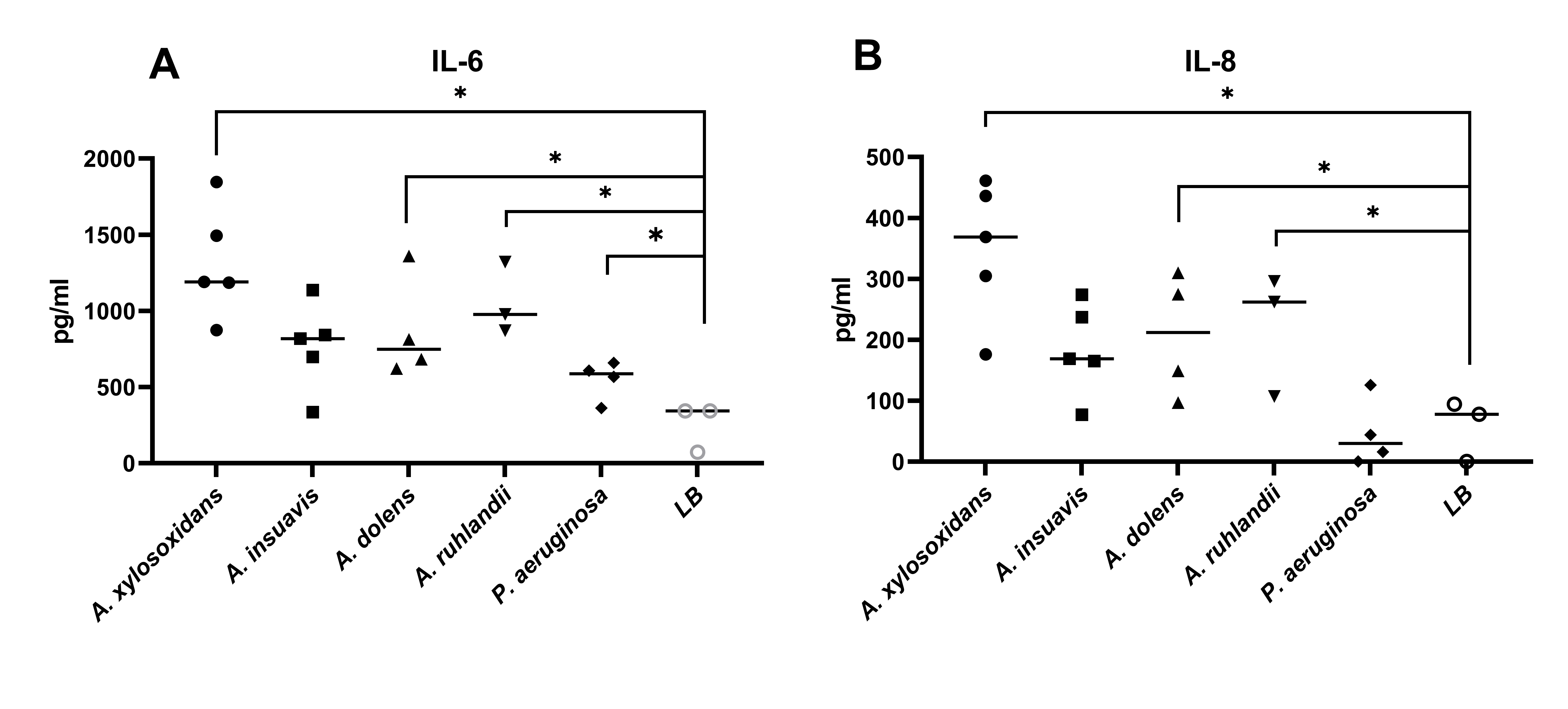

Supplement: Supplemental file 5 — Fig. S3. Download spectrum.00195-23-s0004.tif, TIF file, 0.8 MB [file spectrum.00195-23-s0004.tif]

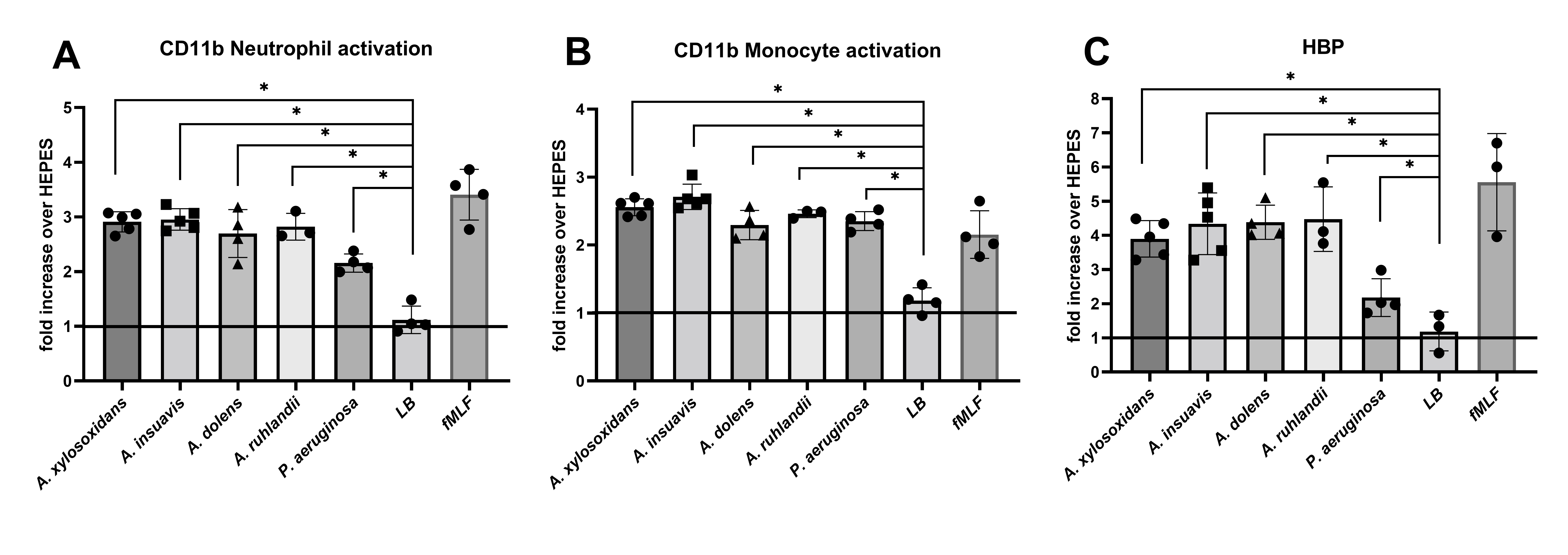

Supplement: Supplemental file 6 — Fig. S4. Download spectrum.00195-23-s0005.tif, TIF file, 1.0 MB [file spectrum.00195-23-s0005.tif]
