# Supplementary material for: Exoproducts of the Most Common Achromobacter Species in Cystic Fibrosis Evoke Similar Inflammatory Responses In Vitro
Source: Microbiol Spectr. 2023 Jun 7;11(4):e00195-23. doi: 10.1128/spectrum.00195-23 (PMC10434066; doi:10.1128/spectrum.00195-23)
Supplement: Supplemental file 1 — File S1. Download spectrum.00195-23-s0006.docx, DOCX file, 0.02 MB [file spectrum.00195-23-s0006.docx]

**1373 – Forward**
AAAAGGAGGGAAGGCGGCGAATGGGACCCTGTTCTCGCCGTCCGACTGCCCCGACCTGCACGACAAGTACGGCAAGGCCTTCGAAGAAGCCTACGTCGGCTATGAAGCGCGCGTCGCCAGCGGCGACCTGAAGCTGTTCAAGAAGATGCCGGCGCTGACCCTGTGGCGCAAGATGCTGTCGATGCTGTTCGAAACCGGCCACCCGTGGATCACGTTCAAGGATCCGTGCAACATCCGTTCGCCGCAGCAGCACGTCGGTGTGGTCCACAGCTCGAATCTGTGCACCGAGATCACGCTGAACACCAACGAATCGGAAATCGCGGTTTGCAACCTGGGGTCGGTGAACCTGGTCGCGCACATGAAGCCGGCCGCTGGCGGCGGTTTCGAACTCGACCACGAGAAGATCAAGCGCACCGTCAGCATCGCCATGCGCATGCTCGACAACGTCATCGACATCAACTATTACGCCGTCGACAAGGCCCGCAACTCCAACGCGCGCCATCGTCCGGTGGGCATGGGCATCATGGGCTTCCAGGACTGCCTGCAGATGATGCGCGTGCCGTACGCGTCGCAAGCCGCCGTTGAATTCGCCGACCGCTCGATGGAAGCGGTGTGCTATCACGCCTACTGGGCCTCGAGCCTGCTGGCCGAAGAACGTGGCCGCTATCAATCGTACGAAGGCTCGCTGTGGTCGCGTGGCATCCTGCCGCAAGACACGTTGAAGATGCTGCGTGACGAACGCGGTGGTCATGTCGAGGTCGATGAATCGAGCACCCTCGATTGGGATGCGTTGCGCGCGCGCATCAAACAACATGGCATGCGCAACTCCAATTGCATCGCAATCGCCCCAACCGCGACTATTTCCATATCATTGGTGTATCTGCGTGCATCGAACCCACTTTCCGAACTGAGCCT

**1373 – Reverse**

GAATACACCAATGATATTGGGAAATAGTCGCGGTTGGGGCGATTGCGATGCAATTGGAGTTGCGCATGCCATGTTGTTTGATGCGCGCGCGCAACGCATCCCAATCGAGGGTGCTCGATTCATCGACCTCGACATGACCACCGCGTTCGTCACGCAGCATCTTCAACGTGTCTTGCGGCAGGATGCCACGCGACCACAGCGAGCCTTCGTACGATTGATAGCGGCCACGTTCTTCGGCCAGCAGGCTCGAGGCCCAGTAGGCGTGATAGCACACCGCTTCCATCGAGCGGTCGGCGAATTCAACGGCGGCTTGCGACGCGTACGGCACGCGCATCATCTGCAGGCAGTCCTGGAAGCCCATGATGCCCATGCCCACCGGACGATGGCGCGCGTTGGAGTTGCGGGCCTTGTCGACGGCGTAATAGTTGATGTCGATGACGTTGTCGAGCATGCGCATGGCGATGCTGACGGTGCGCTTGATCTTCTCGTGGTCGAGTTCGAAACCGCCGCCAGCGGCCGGCTTCATGTGCGCGACCAGGTTCACCGACCCCAGGTTGCAAACCGCGATTTCCGATTCGTTGGTGTTCAGCGTGATCTCGGTGCACAGATTCGAGCTGTGGACCACACCGACGTGCTGCTGCGGCGAACGGATGTTGCACGGATCCTTGAACGTGATCCACGGGTGGCCGGTTTCGAACAGCATCGACAGCATCTTGCGCCACAGGGTCAGCGCCGGCATCTTCTTGAACAGCTTCAGGTCGCCGCTGGCGACGCGCGCTTCATAGCCGACGTAGGCTTCTTCGAAGGCCTTGCCGTACTTGTCGTGCAGGTCGGGGCAGTCGGACGGCGAGAACAGGGTCCATTCGCCGCCTTCCATGACGCGCTTCATGAACAGTCG

PubMLST (Achromobacter) **species:** *A. xylosoxidans* [n=36]

**1191 – Forward**
AGGAAGCGGGCGAATGGGACCCTGTTCTCGCCGTCCGACTGCCCCGACCTGCACGACAAGTACGGCAA

GGCCTTCGAAGAAGCCTACGTCGGCTATGAAGCGCGCGTCGCCAGCGGCGACCTGAAGCTGTTCAAGAAGATGCCGGCGCTGACCCTGTGGCGCAAGATGCTGTCGATGCTGTTCGAAACCGGCCACCCGTGGATCACGTTCAAGGATCCGTGCAACATCCGTTCGCCGCAGCAGCACGTCGGTGTGGTCCACAGCTCGAACCTGTGCACCGAGATCACGCTGAACACCAACGAATCCGAAATCGCGGTTTGCAACCTGGGTTCGGTGAACCTGGTCGCGCACATGAAGCCGGCCGCTGGCGGCGGTTTCGAACTCGACCACGAGAAGATCAAGCGCACCGTCAGCATCGCCATGCGCATGCTCGACAACGTCATCGACATCAACTATTACGCCGTCGACAAGGCCCGCAACTCCAACGCGCGCCATCGCCCGGTGGGCATGGGCATCATGGGCTTCCAGGACTGCCTGCAGATGATGCGCGTGCCGTACGCGTCGCAAGCCGCCGTTGAATTCGCCGACCGCTCGATGGAAGCGGTGTGCTATCACGCCTACTGGGCCTCGAGCCTGCTGGCCGAAGAACGTGGCCGCTATCAATCGTACGAAGGCTCGCTGTGGTCGCGTGGCATCCTGCCGCAAGACACGTTGAAGATGCTGCGTGACGAACGCGGTGGTCATGTCGAGGTCGATGAATCGAGCACGCTCGATTGGGATGCGTTGCGCGCGCGCATCAAACAACATGGCATGCGCAACTCCAATTGCATCGCAATCGCCCCAACCGCGACTATTTCCAATATCATTGGTGTATCTGCGTGCATCGAACCCACTTTCCAGAACTGAGCCCAAAAAAAAACAAAAAAA

**1191 – Reverse**

TCGAGCCGCAGATACACCAATGATATTGGGAAATAGTCGCGGTTGGGGCGATTGCGATGCAATTGGAGTTGCGCATGCCATGTTGTTTGATGCGCGCGCGCAACGCATCCCAATCGAGCGTGCTCGATTCATCGACCTCGACATGACCACCGCGTTCGTCACGCAGCATCTTCAACGTGTCTTGCGGCAGGATGCCACGCGACCACAGCGAGCCTTCGTACGATTGATAGCGGCCACGTTCTTCGGCCAGCAGGCTCGAGGCCCAGTAGGCGTGATAGCACACCGCTTCCATCGAGCGGTCGGCGAATTCAACGGCGGCTTGCGACGCGTACGGCACGCGCATCATCTGCAGGCAGTCCTGGAAGCCCATGATGCCCATGCCCACCGGGCGATGGCGCGCGTTGGAGTTGCGGGCCTTGTCGACGGCGTAATAGTTGATGTCGATGACGTTGTCGAGCATGCGCATGGCGATGCTGACGGTGCGCTTGATCTTCTCGTGGTCGAGTTCGAAACCGCCGCCAGCGGCCGGCTTCATGTGCGCGACCAGGTTCACCGAACCCAGGTTGCAAACCGCGATTTCGGATTCGTTGGTGTTCAGCGTGATCTCGGTGCACAGGTTCGAGCTGTGGACCACACCGAC

GTGCTGCTGCGGCGAACGGATGTTGCACGGATCCTTGAACGTGATCCACGGGTGGCCGGTTTCGAACAGCATCGACAGCATCTTGCGCCACAGGGTCAGCGCCGGCATCTTCTTGAACAGCTTCAGGTCGCCGCTGGCGACGCGCGCTTCATAGCCGACGTAGGCTTCTTCGAAGGCCTTGCCGTACTTGTCGTGCAGGTCGGGGCAGTCGGACGGCGAGAACAGGGTCCATTCGCCGCCTTCCATGACGCGCTTCATGAACAGGCGG

PubMLST (Achromobacter) **species:** *A. xylosoxidans* [n=12]

**1362 – Forward**
GAGGGAAGGCGGCGAATGGGACCCTGTTCTCGCCGTCCGACTGCCCCGACCTGCACGACAAGTACGGC

AAGGCCTTCGAAGAAGCCTACGTCGGCTATGAAGCGCGCGTCGCCAGCGGCGACCTGAAGCTGTTCAAGAAGATGCCGGCGCTGACCCTGTGGCGCAAGATGCTGTCGATGCTGTTCGAAACCGGCCACCCGTGGATCACGTTCAAGGATCCGTGCAACATCCGTTCGCCGCAGCAGCACGTCGGTGTGGTCCACAGCTCGAACCTGTGCACCGAGATCACGCTGAACACCAACGAATCGGAAATCGCGGTTTGCAACCTGGGGTCGGTGAACCTGGTCGCGCACATGAAGCCGGCCGCTGGCGGCGGTTTCGAACTCGACCACGAGAAGATCAAGCGCACCGTCAGCATCGCCATGCGCATGCTCGACAACGTCATCGACATCAACTATTACGCCGTCGACAAGGCCCGCAACTCCAACGCGCGCCATCGCCCGGTGGGCATGGGCATCATGGGCTTCCAGGACTGCCTGCAGATGATGCGCGTGCCGTACGCGTCGCAAGCCGCCGTTGAATTCGCCGACCGCTCGATGGAAGCGGTGTGCTATCACGCCTACTGGGCCTCGAGCCTGCTGGCCGAAGAACGTGGCCGCTATCAATCGTACGAAGGCTCGCTGTGGTCGCGTGGCATCCTGCCGCAAGACACGTTGAAGATGCTGCGTGACGAACGCGGTGGTCATGTCGAGGTCGATGAATCGAGCACGCTCGATTGGGATGCGTTGCGCGCGCGCATCAAACAACATGGCATGCGCAACTCCAATTGCATCGCAATCGCCCCAACCGCGACTATTTCCAATATCATTGGTGTATCTGCGTGCATCGAACCCACTTTCAGAACTGA

**1362 – Reverse**

CGCAGATACACCAATGATATTGGGAAATAGTCGCGGTTGGGGCGATTGCGATGCAATTGGAGTTGCGCATGCCATGTTGTTTGATGCGCGCGCGCAACGCATCCCAATCGAGCGTGCTCGATTCATCGACCTCGACATGACCACCGCGTTCGTCACGCAGCATCTTCAACGTGTCTTGCGGCAGGATGCCACGCGACCACAGCGAGCCTTCGTACGATTGATAGCGGCCACGTTCTTCGGCCAGCAGGCTCGAGGCCCAGTAGGCGTGATAGCACACCGCTTCCATCGAGCGGTCGGCGAATTCAACGGCGGCTTGCGACGCGTACGGCACGCGCATCATCTGCAGGCAGTCCTGGAAGCCCATGATGCCCATGCCCACCGGGCGATGGCGCGCGTTGGAGTTGCGGGCCTTGTCGACGGCGTAATAGTTGATGTCGATGACGTTGTCGAGCATGCGCATGGCGATGCTGACGGTGCGCTTGATCTTCTCGTGGTCGAGTTCGAAACCGCCGCCAGCGGCCGGCTTCATGTGCGCGACCAGGTTCACCGACCCCAGGTTGCAAACCGCGATTTCCGATTCGTTGGTGTTCAGCGTGATCTCGGTGCACAGGTTCGAGCTGTGGACCACACCGACGTGCTGCTGCGGCGAACGGATGTTGCACGGATCCTTGAACGTGATCCACGGGTGGCCGGTTTCGAACAGCATCGACAGCATCTTGCGCCACAGGGTCAGCGCCGGCATCTTCTTGAACAGCTTCAGGTCGCCGCTGGCGACGCGCGCTTCATAGCCGACGTAGGCTTCTTCGAAGGCCTTGCCGTACTTGTCGTGCAGGTCGGGGCAGTCGGACGGCGAGAACAGGGTCCATTCGCCGCCTTCCATGACGCGCTTCATGACAGCGGAAAA

PubMLST (Achromobacter) **species:** *A. xylosoxidans* [n=408]

**1267 – Forward**

CGGGAAGGCGGCGAATGGACCCTGTTCTCGCCGTCCGACTGCCCCGACCTGCACGACAAGTACGGCAAGGCCTTCGAAGAAGCCTACGTCGGCTATGAAGCGCGCGTCGCCAGCGGCGACCTGAAGCTGTTCAAGAAGATGCCGGCGCTGACCCTGTGGCGCAAGATGCTGTCGATGCTGTTCGAAACCGGCCACCCGTGGATCACGTTCAAGGATCCGTGCAACATCCGTTCGCCGCAGCAGCACGTCGGTGTGGTCCACAGCTCGAACCTGTGCACCGAGATCACGCTGAACACCAACGAATCCGAAATCGCGGTTTGCAACCTGGGTTCGGTGAACCTGGTCGCGCACATGAAGCCGGCCGCTGGCGGCGGTTTCGAACTCGACCACGAGAAGATCAAGCGCACCGTCAGCATCGCCATGCGCATGCTCGACAACGTCATCGACATCAACTATTACGCCGTCGACAAGGCCCGCAACTCCAACGCGCGCCATCGCCCGGTGGGCATGGGCATCATGGGCTTCCAGGACTGCCTCCAGATGATGCGCGTGCCGTACGCGTCGCAAGCCGCCGTTGAATTCGCCGACCGCTCGATGGAAGCGGTGTGCTATCACGCCTACTGGGCCTCGAGCCTGCTGGCCGAAGAACGTGGCCGCTATCAATCGTACGAAGGCTCGCTGTGGTCGCGTGGCATCCTGCCGCAAGACACGTTGAAGATGCTGCGTGACGAACGCGGTGGTCATGTCGAGGTCGATGAATCGAGCACGCTCGATTGGGATGCGTTGCGCGCGCGCATCAAACAACATGGCATGCGCAACTCCAATTGCATCGCAATCGCCCCAACCGCGACTATTTCCAATATCATTGGTGTATCTGCGTGCATCGAACCCACTTTCCAGAACTTGACGT

**1267 – Reverse**

TATGCCGCAGATACACCAATGATATTGGGAAATAGTCGCGGTTGGGGCGATTGCGATGCAATTGGAGTTGCGCATGCCATGTTGTTTGATGCGCGCGCGCAACGCATCCCAATCGAGCGTGCTCGATTCATCGACCTCGACATGACCACCGCGTTCGTCACGCAGCATCTTCAACGTGTCTTGCGGCAGGATGCCACGCGACCACAGCGAGCCTTCGTACGATTGATAGCGGCCACGTTCTTCGGCCAGCAGGCTCGAGGCCCAGTAGGCGTGATAGCACACCGCTTCCATCGAGCGGTCGGCGAATTCAACGGCGGCTTGCGACGCGTACGGCACGCGCATCATCTGGAGGCAGTCCTGGAAGCCCATGATGCCCATGCCCACCGGGCGATGGCGCGCGTTGGAGTTGCGGGCCTTGTCGACGGCGTAATAGTTGATGTCGATGACGTTGTCGAGCATGCGCATGGCGATGCTGACGGTGCGCTTGATCTTCTCGTGGTCGAGTTCGAAACCGCCGCCAGCGGCCGGCTTCATGTGCGCGACCAGGTTCACCGAACCCAGGTTGCAAACCGCGATTTCGGATTCGTTGGTGTTCAGCGTGATCTCGGTGCACAGGTTCGAGCTGTGGACCACACCGACG

TGCTGCTGCGGCGAACGGATGTTGCACGGATCCTTGAACGTGATCCACGGGTGGCCGGTTTCGAACAGCATCGACAGCATCTTGCGCCACAGGGTCAGCGCCGGCATCTTCTTGAACAGCTTCAGGTCGCCGCTGGCGACGCGCGCTTCATAGCCGACGTAGGCTTCTTCGAAGGCCTTGCCGTACTTGTCGTGCAGGTCGGGGCAGTCGGACGGCGAGAACAGGGTCCATTCGCCGCCTTCCATGACGCGCTTCATGACAGCGAAG

PubMLST (Achromobacter) **species:** *A. xylosoxidans* [n=1]

**0880 – Forward**

GTTGGAAGGCGGCGAATGGGACCCTGTTCTCGCCGTCCGACTGCCCCGACCTGCACGACAAGTACGGCAAGGCCTTCGAAGAAGCCTACGTCGGCTATGAAGCGCGCGTTGCCAGCGGCGACCTGAAGCTGTTCAAGAAGATGCCGGCGCTGAACCTGTGGCGCAAGATGCTGTCGATGCTGTTCGAAACCGGCCACCCGTGGATCACGTTCAAGGATCCGTGCAACATCCGTTCGCCGCAGCAGCACGTCGGTGTGGTCCACAGCTCGAACCTGTGCACCGAGATCACCCTGAACACCAACGAATCGGAAATCGCGGTTTGCAACCTGGGTTCGGTGAACCTGGTTGCCCACATGAAGCCCGCCGCTGGCGGCGGTTTCGAACTCGACCACGACAAGATCAAGCGCACCGTCAGCATCGCCATGCGCATGCTCGACAACGTCATCGACATCAACTACTACGCCGTCGAGAAGGCCCGCAACTCCAATGCGCGCCATCGTCCGGTGGGCATGGGCATCATGGGTTTCCAGGACTGCCTGCAGATGATGCGCGTGCCGTACGCGTCGCACGCCGCCGTCGAATTCGCCGACACCTCGATGGAAGCCGTGTGCTATCACGCCTACTGGGCCTCGAGCCTGCTGGCTGAAGAACGTGGTCGCTATCAATCGTACGAAGGCTCGCTGTGGTCGCGTGGCATCCTGCCGCAAGACACGTTGAAGATGCTGCGTGACGAACGCGGTGGTCATGTCGAGGTCGATGAATCGAGCACGCTCGATTGGGATGCGTTGCGCGCGCGCATCAATCAACACGGCATGCGCAACTCCAATTGCATCGCAATCGCCCCAACCGCGACTATTTCCAATATCATTGGTGTATCTGCGTGCATCGAACCCACTTTCCAGAACTGAGTA

**0880 – Reverse**

AGTTGAGCCGCAGAATACACCAATGATATTGGGAAATAGTCGCGGTTGGGGCGATTGCGATGCAATTGGAGTTGCGCATGCCGTGTTGATTGATGCGCGCGCGCAACGCATCCCAATCGAGCGTGCTCGATTCATCGACCTCGACATGACCACCGCGTTCGTCACGCAGCATCTTCAACGTGTCTTGCGGCAGGATGCCACGCGACCACAGCGAGCCTTCGTACGATTGATAGCGACCACGTTCTTCAGCCAGCAGGCTCGAGGCCCAGTAGGCGTGATAGCACACGGCTTCCATCGAGGTGTCGGCGAATTCGACGGCGGCGTGCGACGCGTACGGCACGCGCATCATCTGCAGGCAGTCCTGGAAACCCATGATGCCCATGCCCACCGGACGATGGCGCGCATTGGAGTTGCGGGCCTTCTCGACGGCGTAGTAGTTGATGTCGATGACGTTGTCGAGCATGCGCATGGCGATGCTGACGGTGCGCTTGATCTTGTCGTGGTCGAGTTCGAAACCGCCGCCAGCGGCGGGCTTCATGTGGGCAACCAGGTTCACCGAACCCAGGTTGCAAACCGCGATTTCCGATTCGTTGGTGTTCAGGGTGATCTCGGTGCACAGGTTCGAGCTGTGGACCACACC

GACGTGCTGCTGCGGCGAACGGATGTTGCACGGATCCTTGAACGTGATCCACGGGTGGCCGGTTTCGAACAGCATCGACAGCATCTTGCGCCACAGGTTCAGCGCCGGCATCTTCTTGAACAGCTTCAGGTCGCCGCTGGCAACGCGCGCTTCATAGCCGACGTAGGCTTCTTCGAAGGCCTTGCCGTACTTGTCGTGCAGGTCGGGGCAGTCGGACGGCGAGAACAGGGTCCATTCGCCGCCTTCCATGACGCGCTTCATGAACAGGTCGGAAAATCCC

PubMLST (Achromobacter) **species:** *A. insuavis* [n=3]

**0714 – Forward**

TGGGAAGGCGGCGAATGGACCCTCTTCTCGCCGTCCGACTGCCCCGACCTGCACGACAAGTACGGCAAGGCCTTCGAAGAAGCCTACGTCGGCTATGAAGCGCGCGTCGCCAGCGGCGACCTGAAGCTGTTCAAGAAGATGCCGGCGCTGACGCTGTGGCGCAAGATGCTGTCGATGCTGTTCGAAACCGGCCACCCGTGGATCACGTTCAAGGATCCGTGCAACATCCGTTCGCCGCAGCAGCACGTCGGTGTGGTCCACAGCTCGAACCTGTGCACCGAGATCACGCTGAACACCAACGAATCGGAAATCGCGGTTTGCAACCTGGGTTCGGTGAACCTGGTCGCGCACATGAAGCCCGCCGCCGGCGGCGGTTTCGAACTCGACCACGACAAGATCAAGCGCACCGTCAGCATCGCCATGCGCATGCTCGACAACGTCATCGACATCAACTACTACGCCGTCGAGAAGGCCCGCAACTCCAATGCGCGCCATCGTCCGGTGGGCATGGGCATCATGGGTTTCCAGGACTGCCTGCAGATGATGCGCGTGCCGTACGCGTCGCACGCCGCCGTCGAATTCGCCGACACCTCGATGGAAGCCGTGTGCTATCACGCCTACTGGGCCTCGAGCCTGCTGGCTGAAGAACGTGGTCGCTATCAATCGTACGAAGGCTCGCTGTGGTCGCGTGGCATCCTGCCGCAAGACACGTTGAAGATGCTGCGTGACGAACGCGGTGGTCATGTCGAGGTCGATGAATCGAGCACGCTCGATTGGGATGCGTTGCGCGCGCGCATCAATCAACACGGCATGCGCAACTCCAATTGCATCGCAATCGCCCCAACCGCGACTATTTCCAATATCATTGGTGTATCTGCGTGCATCGAACCCACTTTCCAGAACTGAGCCCCAAA

**0714 – Reverse**

CGATGCACGCAGATACACCAATGATATTGGGAAATAGTCGCGGTTGGGGCGATTGCGATGCAATTGGAGTTGCGCATGCCGTGTTGATTGATGCGCGCGCGCAACGCATCCCAATCGAGCGTGCTCGATTCATCGACCTCGACATGACCACCGCGTTCGTCACGCAGCATCTTCAACGTGTCTTGCGGCAGGATGCCACGCGACCACAGCGAGCCTTCGTACGATTGATAGCGACCACGTTCTTCAGCCAGCAGGCTCGAGGCCCAGTAGGCGTGATAGCACACGGCTTCCATCGAGGTGTCGGCGAATTCGACGGCGGCGTGCGACGCGTACGGCACGCGCATCATCTGCAGGCAGTCCTGGAAACCCATGATGCCCATGCCCACCGGACGATGGCGCGCATTGGAGTTGCGGGCCTTCTCGACGGCGTAGTAGTTGATGTCGATGACGTTGTCGAGCATGCGCATGGCGATGCTGACGGTGCGCTTGATCTTGTCGTGGTCGAGTTCGAAACCGCCGCCGGCGGCGGGCTTCATGTGCGCGACCAGGTTCACCGAACCCAGGTTGCAAACCGCGATTTCCGATTCGTTGGTGTTCAGCGTGATCTCGGTGCACAGGTTCGAGCTGTGGACCACACCGACGTGCTGCTGCGGCGAACGGATGTTGCACGGATCCTTGAACGTGATCCACGGGTGGCCGGTTTCGAACAGCATCGACAGCATCTTGCGCCACAGCGTCAGCGCCGGCATCTTCTTGAACAGCTTCAGGTCGCCGCTGGCGACGCGCGCTTCATAGCCGACGTAGGCTTCTTCGAAGGCCTTGCCGTACTTGTCGTGCAGGTCGGGGCAGTCGGACGGCGAGAAGAGGGTCCATTCGCCGCCTTCCATGACGCGCTTCATGACAGCGG

PubMLST (Achromobacter) **species:** *A. insuavis* [n=24]

**1721 – Forward**

﻿CGGAAGGCGGCGAATGGACCTGTTCTCGCCGTCCGACTGCCCCGACCTGCACGACAAGTACGGCAAGGCCTTCGAAGAAGCCTACGTCGGCTATGAAGCGCGCGTCGCCAGCGGCGACCTGAAGCTGTTCAAGAAGATGCCGGCGCTGACGCTGTGGCGCAAGATGCTGTCGATGCTGTTCGAAACCGGCCACCCGTGGATCACGTTCAAGGATCCGTGCAACATCCGTTCGCCGCAGCAGCACGTCGGTGTGGTCCACAGCTCGAACCTGTGCACCGAGATCACGCTGAACACCAACGAATCGGAAATCGCGGTTTGCAACCTGGGTTCGGTGAACCTGGTCGCGCACATGAAGCCCGCCGCCGGCGGCGGTTTCGAACTCGACCACGACAAGATCAAGCGCACCGTCAGCATCGCCATGCGCATGCTCGACAACGTCATCGACATCAACTACTACGCCGTCGAGAAGGCCCGCAACTCCAATGCGCGCCATCGTCCGGTGGGCATGGGCATCATGGGTTTCCAGGACTGCCTGCAGATGATGCGCGTGCCGTACGCGTCGCACGCCGCCGTCGAATTCGCCGACACCTCGATGGAAGCCGTGTGCTATCACGCCTACTGGGCCTCGAGCCTGCTGGCTGAAGAACGTGGTCGCTATCAATCGTACGAAGGCTCGCTGTGGTCGCGTGGCATCCTGCCGCAAGACACGTTGAAGATGCTGCGTGACGAACGCGGTGGTCATGTCGAGGTCGATGAATCGAGCACCCTCGATTGGGATGCGTTGCGCGCGCGCATCAATCAACACGGCATGCGCAACTCCAATTGCATCGCAATCGCCCCAACCGCGACTATTTCCAATATCATTGGTGTATCTGCGTGCATCGAACCCACTTTCCAGAACTTGAGCCCCAA

**1721 – Reverse**

﻿AGTCGATGCCGCAGAATACACCAATGATATTGGAAATAGTCGCGGTTGGGGCGATTGCGATGCAATTGGAGTTGCGCATGCCGTGTTGATTGATGCGCGCGCGCAACGCATCCCAATCGAGGGTGCTCGATTCATCGACCTCGACATGACCACCGCGTTCGTCACGCAGCATCTTCAACGTGTCTTGCGGCAGGATGCCACGCGACCACAGCGAGCCTTCGTACGATTGATAGCGACCACGTTCTTCAGCCAGCAGGCTCGAGGCCCAGTAGGCGTGATAGCACACGGCTTCCATCGAGGTGTCGGCGAATTCGACGGCGGCGTGCGACGCGTACGGCACGCGCATCATCTGCAGGCAGTCCTGGAAACCCATGATGCCCATGCCCACCGGACGATGGCGCGCATTGGAGTTGCGGGCCTTCTCGACGGCGTAGTAGTTGATGTCGATGACGTTGTCGAGCATGCGCATGGCGATGCTGACGGTGCGCTTGATCTTGTCGTGGTCGAGTTCGAAACCGCCGCCGGCGGCGGGCTTCATGTGCGCGACCAGGTTCACCGAACCCAGGTTGCAAACCGCGATTTCCGATTCGTTGGTGTTCAGCGTGATCTCGGTGCACAGGTTCGAGCTGTGGACCACACCGACGTGCTGCTGCGGCGAACGGATGTTGCACGGATCCTTGAACGTGATCCACGGGTGGCCGGTTTCGAACAGCATCGACAGCATCTTGCGCCACAGCGTCAGCGCCGGCATCTTCTTGAACAGCTTCAGGTCGCCGCTGGCGACGCGCGCTTCATAGCCGACGTAGGCTTCTTCGAAGGCCTTGCCGTACTTGTCGTGCAGGTCGGGGCAGTCGGACGGCGAGAACAGGGTCCATTCGCCGCCTTCCATGACGCGCTTCATGAACAGGTCGAGAA

PubMLST (Achromobacter) **species:** *A. insuavis* [n=18]

**0753 – Forward**
AAAGGTGGGAAGGCGGCGAATGGGACCCTGTTCTCGCCGTCCGACTGCCCCGACCTGCACGACAAGTACGGCAAGGCCTTCGAAGAAGCCTACGTCGGCTATGAAGCGCGCGTTGCCAGCGGCGACCTGAAGCTGTTCAAGAAGATGCCGGCGCTGACCCTGTGGCGCAAGATGCTGTCGATGCTGTTCGAAACCGGCCACCCGTGGATCACGTTCAAGGATCCGTGCAACATCCGTTCGCCGCAGCAGCACGTCGGTGTGGTCCACAGCTCGAACCTGTGCACCGAGATCACGCTGAACACCAACGAATCCGAAATCGCGGTTTGCAACCTGGGTTCGGTGAACCTGGTCGCGCACATGAAGCCGGCCGCTGGCGGCGGTTTCGAACTCGACCACGAGAAGATCAAGCGCACCGTCAGCATCGCCATGCGCATGCTCGACAACGTCATCGACATCAACTATTACGCCGTCGACAAGGCCCGCAACTCCAACGCGCGCCATCGTCCGGTGGGCATGGGCATCATGGGTTTCCAGGACTGCCTGCAGATGATGCGCGTGCCGTACGCATCGCAAGCCGCCGTCGAATTCGCCGATCGTTCGATGGAAGCGGTGTGCTATCACGCCTACTGGGCCTCGAGCCTGCTGGCCGAAGAACGTGGCCGCTATCAATCGTACGAAGGCTCGCTGTGGTCGCGTGGCATCCTGCCGCAAGACACGTTGAAGATGCTGCGTGACGAACGCGGCGGTCATGTCGAGGTCGATGAATCGAGCACGCTCGATTGGGATGCGTTGCGCGCGCGCATCAAACAACATGGCATGCGCAACTCCAATTGCATCGCAATCGCCCCAACCGCGACTATTTCCAATATCATTGGTGTATCTGCGTGCATCGAACCCACTTTCCAGAACTGTAGCTC

**0753 – Reverse**

AAGGTCGAGCCGCAGAATACACCAATGATATTGGGAAATAGTCGCGGTTGGGGCGATTGCGATGCAATTGGAGTTGCGCATGCCATGTTGTTTGATGCGCGCGCGCAACGCATCCCAATCGAGCGTGCTCGATTCATCGACCTCGACATGACCGCCGCGTTCGTCACGCAGCATCTTCAACGTGTCTTGCGGCAGGATGCCACGCGACCACAGCGAGCCTTCGTACGATTGATAGCGGCCACGTTCTTCGGCCAGCAGGCTCGAGGCCCAGTAGGCGTGATAGCACACCGCTTCCATCGAACGATCGGCGAATTCGACGGCGGCTTGCGATGCGTACGGCACGCGCATCATCTGCAGGCAGTCCTGGAAACCCATGATGCCCATGCCCACCGGACGATGGCGCGCGTTGGAGTTGCGGGCCTTGTCGACGGCGTAATAGTTGATGTCGATGACGTTGTCGAGCATGCGCATGGCGATGCTGACGGTGCGCTTGATCTTCTCGTGGTCGAGTTCGAAACCGCCGCCAGCGGCCGGCTTCATGTGCGCGACCAGGTTCACCGAACCCAGGTTGCAAACCGCGATTTCGGATTCGTTGGTGTTCAGCGTGATCTCGGTGCACAGGTTCGAGCTGTGGACCACA

CCGACGTGCTGCTGCGGCGAACGGATGTTGCACGGATCCTTGAACGTGATCCACGGGTGGCCGGTTTCGAACAGCATCGACAGCATCTTGCGCCACAGGGTCAGCGCCGGCATCTTCTTGAACAGCTTCAGGTCGCCGCTGGCAACGCGCGCTTCATAGCCGACGTAGGCTTCTTCGAAGGCCTTGCCGTACTTGTCGTGCAGGTCGGGGCAGTCGGACGGCGAGAACAGGGTCCATTCGCCGCCTTCCATGACGCGCTTCATGAACAGTCGGAAAATCCC

PubMLST (Achromobacter) **species:** *A. dolens* [n=45]

**1201 – Forward**

CTGGAAGGCGGCGAATGGGACCCTGTTCTCGCCGTCCGACTGCCCCGACCTGCACGACAAGTACGGCAAGGCCTTCGAAGAAGCCTATGTCGGCTATGAAGCGCGCGTTGCCAGCGGCGACCTGAAGCTGTTCAAGAAGATGCCGGCGCTGACCCTGTGGCGCAAGATGCTGTCGATGCTGTTCGAAACCGGCCACCCGTGGATCACGTTCAAGGATCCGTGCAACATCCGTTCGCCGCAGCAGCACGTCGGTGTGGTCCACAGCTCGAACCTGTGCACCGAGATCACGCTGAACACCAACGAATCCGAAATCGCGGTTTGCAACCTGGGTTCGGTGAACCTGGTCGCGCACATGAAGCCGGCCGCTGGCGGCGGTTTCGAACTCGACCACGAGAAGATCAAGCGCACCGTCAGCATCGCCATGCGCATGCTCGACAACGTCATCGACATCAACTATTACGCCGTCGACAAGGCCCGCAACTCCAACGCGCGCCATCGTCCGGTGGGCATGGGCATCATGGGTTTCCAGGACTGCCTGCAGATGATGCGCGTGCCGTACGCATCGCAAGCCGCCGTCGAATTCGCCGATCGTTCGATGGAAGCGGTGTGCTATCACGCCTACTGGGCCTCGAGCCTGCTGGCCGAAGAACGTGGCCGCTATCAATCGTACGAAGGCTCGCTGTGGTCGCGTGGCATCCTGCCGCAAGACACGTTGAAGATGCTGCGTGACGAACGCGGCGGTCATGTCGAGGTCGATGAATCGAGCACGCTCGATTGGGATGCGTTGCGCGCGCGCATCAAACAACATGGCATGCGCAACTCCAATTGCATCGCAATCGCCCCAACCGCGACTATTTCCAATATCATTGGTGTATCTGCGTGCATCGAACCCACTTTCCAGAACTGAGCTCC

**1201 – Reverse**

AAAGGTCGAGGCCGCAGATACACCAATGATATTGGGAAATAGTCGCGGTTGGGGCGATTGCGATGCAATTGGAGTTGCGCATGCCATGTTGTTTGATGCGCGCGCGCAACGCATCCCAATCGAGCGTGCTCGATTCATCGACCTCGACATGACCGCCGCGTTCGTCACGCAGCATCTTCAACGTGTCTTGCGGCAGGATGCCACGCGACCACAGCGAGCCTTCGTACGATTGATAGCGGCCACGTTCTTCGGCCAGCAGGCTCGAGGCCCAGTAGGCGTGATAGCACACCGCTTCCATCGAACGATCGGCGAATTCGACGGCGGCTTGCGATGCGTACGGCACGCGCATCATCTGCAGGCAGTCCTGGAAACCCATGATGCCCATGCCCACCGGACGATGGCGCGCGTTGGAGTTGCGGGCCTTGTCGACGGCGTAATAGTTGATGTCGATGACGTTGTCGAGCATGCGCATGGCGATGCTGACGGTGCGCTTGATCTTCTCGTGGTCGAGTTCGAAACCGCCGCCAGCGGCCGGCTTCATGTGCGCGACCAGGTTCACCGAACCCAGGTTGCAAACCGCGATTTCGGATTCGTTGGTGTTCAGCGTGATCTCGGTGCACAGGTTCGAGCTGTGGACCAC

ACCGACGTGCTGCTGCGGCGAACGGATGTTGCACGGATCCTTGAACGTGATCCACGGGTGGCCGGTTTCGAACAGCATCGACAGCATCTTGCGCCACAGGGTCAGCGCCGGCATCTTCTTGAACAGCTTCAGGTCGCCGCTGGCAACGCGCGCTTCATAGCCGACATAGGCTTCTTCGAAGGCCTTGCCGTACTTGTCGTGCAGGTCGGGGCAGTCGGACGGCGAGAACAGGGTCCATTCGCCGCCTTCCATGACGCGCTTCATGAACAGTCGAAAACC

PubMLST (Achromobacter) **species:** *A. dolens* [n=6]

**1017a – Forward**
GTTGGAAGGCGGCGAATGGGACCCTGTTCTCGCCGTCCGACTGCCCCGACCTGCACGACAAGTACGGCAAGGCCTTCGAAGAAGCCTACGTCGGCTATGAAGCGCGCGTCGCCAGCGGCGACCTGAAGCTGTTCAAGAAGATGCCGGCGCTGACCCTGTGGCGCAAGATGCTGTCGATGCTGTTCGAAACCGGCCACCCGTGGATCACGTTCAAGGATCCGTGCAACATCCGTTCGCCGCAGCAGCACGTCGGTGTGGTCCACAGCTCGAACCTGTGCACCGAGATCACGCTGAACACCAACGAATCGGAAATCGCGGTTTGCAACCTGGGTTCGGTGAACCTGGTCGCGCACATGAAGCCGGCCGCTGGCGGCGGTTTCGAACTCGACCACGACAAGATCAAGCGCACCGTCAGCATCGCCATGCGCATGCTCGACAACGTCATCGACATCAACTATTACGCCGTCGACAAGGCCCGCAATTCCAACGCGCGCCATCGTCCGGTGGGCATGGGCATCATGGGTTTCCAGGACTGCCTGCAGATGATGCGCGTGCCGTACGCATCGCAAGCCGCCGTCGAATTCGCCGATCGTTCGATGGAAGCCGTGTGCTATCACGCCTACTGGGCCTCGAGCCTGCTGGCCGAAGAACGTGGCCGCTATCAATCGTACGAAGGCTCGCTGTGGTCGCGTGGCATCCTGCCGCAAGACACGTTGAAGATGCTGCGTGACGAACGCGGTGGTCATGTCGAGGTCGATGAATCGAGCACGCTCGATTGGGATGCGTTGCGCGCGCGCATCAAACAACATGGCATGCGCAACTCCAATTGCATCGCAATCGCCCCAACCGCGACTATTTCCAATATCATTGGTGTATCTGCGTGCATCGAACCCACTTTCCGAACTGAGCCAAAAAAAAAATAAA

**1017a – Reverse**

CAGAATACACCAATGATATTGGGAAATAGTCGCGGTTGGGGCGATTGCGATGCAATTGGAGTTGCGCATGCCATGTTGTTTGATGCGCGCGCGCAACGCATCCCAATCGAGCGTGCTCGATTCATCGACCTCGACATGACCACCGCGTTCGTCACGCAGCATCTTCAACGTGTCTTGCGGCAGGATGCCACGCGACCACAGCGAGCCTTCGTACGATTGATAGCGGCCACGTTCTTCGGCCAGCAGGCTCGAGGCCCAGTAGGCGTGATAGCACACGGCTTCCATCGAACGATCGGCGAATTCGACGGCGGCTTGCGATGCGTACGGCACGCGCATCATCTGCAGGCAGTCCTGGAAACCCATGATGCCCATGCCCACCGGACGATGGCGCGCGTTGGAATTGCGGGCCTTGTCGACGGCGTAATAGTTGATGTCGATGACGTTGTCGAGCATGCGCATGGCGATGCTGACGGTGCGCTTGATCTTGTCGTGGTCGAGTTCGAAACCGCCGCCAGCGGCCGGCTTCATGTGCGCGACCAGGTTCACCGAACCCAGGTTGCAAACCGCGATTTCCGATTCGTTGGTGTTCAGCGTGATCTCGGTGCACAGGTTCGAGCTGTGGACCACACCGACGTGCTGC

TGCGGCGAACGGATGTTGCACGGATCCTTGAACGTGATCCACGGGTGGCCGGTTTCGAACAGCATCGACAGCATCTTGCGCCACAGGGTCAGCGCCGGCATCTTCTTGAACAGCTTCAGGTCGCCGCTGGCGACGCGCGCTTCATAGCCGACGTAGGCTTCTTCGAAGGCCTTGCCGTACTTGTCGTGCAGGTCGGGGCAGTCGGACGGCGAGAACAGGGTCCATTCGCCGCCTTCCATGACGCGCTTCATGAACAGGTCGAAA

PubMLST (Achromobacter) **species:** *A. ruhlandii* [n=24]

Source: <https://pubmlst.org/bigsdb?db=pubmlst_achromobacter_seqdef&page=sequenceQuery>

Accessed 2023-05-03
